# The impact of psychological nursing interventions on occupational burnout in sterilization supply center personnel: A retrospective analysis

**DOI:** 10.1097/MD.0000000000043831

**Published:** 2025-08-22

**Authors:** Junli Ma, Shiying Wang, Aibo Yang, Yueting Li, Ying Chen

**Affiliations:** a Department of Central Sterile Supply, The Fourth People’s Hospital of Shanghai, Affiliated to Tongji University, Shanghai, China; b Department of Central Sterile Supply, The Second Affiliated Hospital of Naval Medical University, Shanghai, China; c Department of Nursing, The Fourth People’s Hospital of Shanghai, Affiliated to Tongji University, Shanghai, China.

**Keywords:** anxiety, depression, occupational burnout, psychological care interventions, sterilization supply center

## Abstract

This study investigates the prevalence of occupational burnout among hospital sterilization supply center staff and evaluates the effectiveness of psychological interventions in mitigating burnout and associated mental health concerns, such as anxiety and depression. A retrospective analysis was conducted on 120 sterilization supply center employees, categorized into an intervention group (n = 40) receiving structured psychological support–including stress management, emotional regulation training, and mindfulness practices–and a nonintervention group (n = 80). Burnout levels were assessed using the Maslach Burnout Inventory (MBI), while the self-rating anxiety scale (SAS) and self-rating depression scale (SDS) measured anxiety and depression, respectively. Statistical comparisons (Chi-square, *t*-tests, ANOVA, and regression analyses) were performed to assess intervention outcomes. The intervention group demonstrated marked reductions in burnout metrics: emotional exhaustion decreased from 26.5 ± 8.3 to 24.1 ± 7.9 (*P* = .031), depersonalization declined from 19.2 ± 5.6 to 16.3 ± 5.2 (*P* = .048), and personal accomplishment improved from 35.1 ± 8.4 to 38.3 ± 7.1 (*P* = .042). Concurrently, anxiety scores (SAS) dropped from 39.1 ± 14.7 to 34.2 ± 12.4 (*P* = .033), and depression scores (SDS) fell from 34.4 ± 13.2 to 29.6 ± 11.8 (*P* = .047). Regression analysis confirmed that psychological interventions significantly alleviated emotional exhaustion (β = −2.45, *P* = .031), depersonalization (β = −2.71, *P* = .048), anxiety (β = −4.15, *P* = .024), and depression (β = −4.12, *P* = .027). Targeted psychological care effectively reduces occupational burnout and enhances mental well-being among sterilization supply center staff, particularly those facing high workloads. These findings underscore the value of integrating psychological support into workplace wellness initiatives to improve employee resilience in high-stress healthcare settings.

## 1. Introduction

Burnout refers to a series of psychological and physiological responses, including emotional exhaustion, depersonalization, and a decrease in personal accomplishment, resulting from prolonged exposure to high-pressure work environments, excessive workload, resource shortages, and lack of social support.^[1,2]^ Particularly in high-demand professions such as healthcare, education, and social services, burnout has become a serious issue affecting employees’ physical and mental health, work efficiency, and patient safety.^[3,4]^ In recent years, with increasing work intensity and the growing prominence of mental health issues, burnout has emerged as an important global public health concern.^[5]^

In hospital settings, especially in support departments such as sterilization supply centers, the work-related stress faced by staff is often overlooked. However, these workers may also be at a higher risk for burnout, which can impact their physical and mental health as well as their work quality. Studies have shown that burnout is closely associated with mental health issues such as hypertension, depression, and anxiety, and may lead to decreased work efficiency, increased error rates, and even affect teamwork and patient safety.^[6,7]^ Therefore, understanding the burnout status of sterilization supply center staff and implementing effective interventions to alleviate burnout are crucial for ensuring internal work quality and improving the physical and mental health of employees.

Currently, research on burnout among sterilization supply center staff is limited, with existing studies primarily focusing on the impact of work stress and working hours on burnout. Although some studies have explored the role of psychological nursing interventions in reducing burnout, these studies often have small sample sizes, short-term interventions, and lack long-term effect tracking and evaluation of diverse intervention strategies.^[8,9]^ Moreover, existing research has not fully considered how the specific forms and frequencies of psychological nursing interventions affect the different dimensions of burnout. Therefore, the impact of different psychological intervention methods on burnout dimensions remains to be thoroughly explored.

This study aims to retrospectively analyze the burnout status of sterilization supply center staff and evaluate the effects of psychological nursing interventions on various dimensions of burnout. We hypothesize that staff receiving regular psychological nursing interventions will show significant improvements in emotional exhaustion, depersonalization, and personal accomplishment. Compared to those who did not receive interventions, the intervention group is expected to have better mental health levels and higher job satisfaction.

The innovation of this study lies in its large-scale retrospective analysis of sterilization supply center staff, assessing the long-term effects of different psychological intervention methods on burnout. By comparing the burnout levels of staff who receive regular versus irregular interventions, we will explore the potential of psychological nursing interventions in alleviating burnout. This will provide empirical evidence for the promotion of similar interventions in other high-pressure occupational groups in the future. In summary, this study will not only help fill the gap in current research on burnout among sterilization supply center staff, but also provide hospital management with specific intervention strategies to improve staff physical and mental health, work performance, and enhance overall hospital service quality.

## 2. Research methods

### 2.1. Study design

This study was approved by the Ethics Committee of the Fourth People’s Hospital of Shanghai, affiliated with Tongji University, under the approval number: TJ2023-0425. This is a retrospective study aimed at evaluating the burnout status of sterilization supply center staff in hospitals and examining its relationship with psychological nursing interventions. By reviewing hospital archival data, we will analyze changes in the burnout and mental health status of sterilization supply center staff who received psychological nursing interventions between June 2023 and December 2024. These results will be compared with those of staff who did not receive any interventions. The intervention group will consist of 40 staff members who regularly received at least 1 form of psychological intervention, while the nonintervention group will include 80 staff members who received either irregular interventions or no interventions at all.

Inclusion Criteria: Job type: Staff members working in hospital sterilization supply centers, including positions such as manual workers, disinfectors, and technicians. Age range: Between 18 and 60 years old. Work experience: Must have worked in the hospital sterilization supply center for at least 1 year. Informed consent: Participants must voluntarily agree to participate in the study and sign an informed consent form. Although this is a retrospective study, participants consent to the use of their archival data for research analysis. Burnout score: No specific burnout score range will be set. All staff members working in the sterilization supply center will be included, regardless of their burnout levels. Burnout will be comprehensively assessed using the Maslach Burnout Inventory (MBI)^[10]^ to reflect different levels of burnout. Mental health assessment: Participants must have complete anxiety and depression scores, but individuals with severe preexisting mental health conditions will be excluded. Intervention group criteria: Individuals who have regularly received at least 1 type of psychological nursing intervention (e.g., stress management training, emotional regulation skills, meditation, and mindfulness exercises) for at least 3 months, with a frequency of ≥ 2 times per month. Non-intervention group criteria: Individuals who have not received any psychological nursing interventions or those whose intervention frequency is below the above standard (e.g., occasional participation or receiving nonsystematic interventions).

Exclusion Criteria: Severe physical or mental illness: Individuals diagnosed with severe mental disorders (such as schizophrenia, major depression, bipolar disorder, etc), or those with serious physical conditions (such as cancer, cardiovascular diseases, etc) that may affect the interpretation of the study results. Pregnant or breastfeeding women: Due to the physiological changes during pregnancy or breastfeeding, to avoid interference with the intervention effects. Currently receiving other forms of psychological treatment or medication: Individuals currently undergoing other psychological treatments (such as cognitive behavioral therapy) or taking medications affecting mood (e.g., antidepressants) that could interfere with the accurate assessment of the intervention effects. Significant work changes: Individuals experiencing frequent job changes, long-term leave, or resignation, which may affect the continuity of the intervention and the reliability of the data. Language barriers: Participants who are unable to effectively understand the intervention content or assessment scales, such as those with severe language impairments. History or current suicidal tendencies: Individuals with a history or current risk of suicide, as their inclusion could significantly impact the intervention outcomes and may require specialized treatment.

## 3. Psychological intervention methods

This study involves various psychological intervention methods to assess their impact on the burnout of hospital sterilization supply center staff. The specific psychological interventions include stress management training, emotional regulation skills, meditation and mindfulness practices, cognitive behavioral therapy (CBT), group therapy (support groups), and other types of interventions such as art therapy and relaxation training. An overview of each intervention method is provided below:

### 3.1. *Stress management training*^[11]^

This intervention aims to help staff identify and manage stress generated from work, providing effective coping strategies. The intervention content includes time management, relaxation training, cognitive restructuring, and emotional regulation skills. These strategies are designed to help employees reduce work-related stress through practical techniques. The intervention frequency is 2.5 sessions per month, with the intervention duration being 3 months or longer.

### 3.2. *Emotional regulation skills*^[12]^

This intervention trains staff to identify and regulate emotional responses, particularly in managing negative emotions such as anxiety and anger. The intervention includes emotional recognition, emotional expression techniques, and the cultivation of positive emotions. Such interventions are typically conducted twice per month and aim to enhance employees’ emotional self-regulation skills.

### 3.3. *Meditation and mindfulness practices*^[13]^

This intervention uses meditation and mindfulness training to help staff improve self-awareness and emotional regulation. Through techniques such as breathing exercises, body scanning, and meditation, participants are able to relax both their bodies and minds, reducing anxiety and stress. The intervention is conducted with a frequency of 1.8 sessions per month, typically lasting around 1 hour per session.

### 3.4. *Cognitive behavioral therapy (CBT*)^[14]^

CBT works by altering negative thought patterns and behavioral habits to alleviate psychological issues. For burnout, CBT helps employees identify and adjust irrational thinking patterns, thereby reducing emotional exhaustion and depersonalization. The intervention occurs twice per month and typically lasts for 3 months or more.

### 3.5. *Group therapy (support groups*)^[15]^

This intervention provides social support and psychological counseling through group formats, where participants share experiences and feelings, receiving emotional support and psychological guidance. The intervention is conducted once per month, typically in 1–2 hour group discussion sessions.

### 3.6. *Other interventions (e.g., art therapy, relaxation training*)^[16]^

This category includes interventions such as art creation, music therapy, and relaxation training, aimed at helping employees relax and alleviate work-related stress. These interventions are flexible and typically conducted once per month, with each session lasting between 30 minutes and 1 hour.

All psychological interventions are carried out for at least 3 months, with the intervention frequency adhering to the standards of each specific method to ensure effective management of burnout. The selection of each intervention method is based on the staff’s needs and the feasibility of available hospital resources.

## 4. Data collection

### 4.1. *Demographic information*

In this study, demographic data will be collected, including participants’ gender, age, work experience, position, marital status, whether they have children, educational level, family income, health status, and job satisfaction. Gender will be categorized as male and female, and age will be divided into 4 groups: 18–30 years, 31–40 years, 41–50 years, and 51 years and above. Work experience will be categorized into 3 groups: 1–3 years, 4–6 years, and more than 7 years. Positions will include roles such as manual workers, disinfectors, and technicians. Marital status will be divided into married and unmarried, and having children will be classified as “Yes” or “No.” Educational level will be categorized into “high school or below” and “university or above.” Family income will be classified as low income (≤3000 RMB/month), middle income (3001–6000 RMB/month), and high income (>6000 RMB/month). Health status will be categorized as good, fair, or poor, and job satisfaction will be divided into high, medium, and low levels. These data will be collected through the hospital’s employee management system and questionnaire surveys, ensuring comparability of background characteristics between the intervention and nonintervention groups.

### 4.2. Burnout assessment

Burnout will be assessed using the MBI, which includes 3 dimensions: emotional exhaustion, depersonalization, and personal accomplishment. The emotional exhaustion dimension primarily evaluates feelings of fatigue and emotional depletion in the workplace. The depersonalization dimension measures the extent to which staff exhibit indifference, negativity, and a lack of empathy in their professional roles. The personal accomplishment dimension assesses whether staff feel a sense of self-efficacy, achievement, and fulfillment in their work. The scores for each dimension will be quantified using the MBI, and participants’ burnout levels will be categorized into high, moderate, and low burnout based on their scores. This categorization will facilitate group comparisons in subsequent analyses.

### 4.3. Anxiety and depression assessment

To comprehensively assess participants’ mental health, particularly anxiety and depression symptoms, this study will use standardized self-assessment scales: the self-rating anxiety scale (SAS) and the self-rating depression scale (SDS).^[17,18]^ Anxiety symptoms will be quantified using the SAS to determine staff’s anxiety levels, while depression symptoms will be assessed using the SDS to capture participants’ depressive moods. These assessments will help analyze emotional changes before and after the intervention, providing insight into whether psychological nursing interventions have a positive effect on alleviating anxiety and depression symptoms.

### 4.4. Psychological nursing intervention records

For the intervention group, the study will carefully document the type, frequency, and duration of each participant’s psychological nursing interventions. The intervention types include stress management training, emotional regulation skills, meditation and mindfulness practices, (CBT), group therapy, art therapy, and other forms. All interventions will be conducted by professionally trained psychological nursing staff. The intervention frequency will generally be at least twice per month, with each session lasting no <30 minutes. The duration of the intervention will be at least 3 months to ensure continuity and effectiveness. Recording these intervention data will help assess the specific impact of different psychological interventions on burnout and provide the necessary basis for further analysis.

### 4.5. Other mental health-related data

When collecting mental health-related data, the study will also consider other psychological health factors that may affect burnout. Through hospital records, the study will gather information about participants’ personal history of mental health issues, such as suicidal tendencies or severe mood disorders. These data will help exclude potential confounding variables, ensuring the comparability of mental health backgrounds between the intervention and nonintervention groups and avoiding unnecessary interference from external factors in the study results.

## 5. Statistical analysis

In this study, data statistical analysis will be performed using SPSS statistical software.^[19]^ For comparisons of basic demographic characteristics, Chi-square tests (χ^2^) and independent samples *t*-tests will be used to assess the differences between the intervention and nonintervention groups with respect to variables such as gender, age, work experience, and position. For the evaluation of burnout, anxiety, and depression symptoms, independent samples *t*-tests (*t*-values) will be used to compare the mean scores between the 2 groups. To further analyze the intervention effects, analysis of variance (ANOVA) will be used to compare the differences in emotional exhaustion, depersonalization, and personal accomplishment dimensions of burnout across different psychological intervention methods.

The classification of burnout dimensions will involve Chi-square tests (χ^2^) for intergroup comparisons, evaluating the impact of interventions on the proportion of participants with high and low burnout levels. Correlation analysis will use Pearson’s correlation coefficient (r) to assess the relationship between burnout and anxiety/depression symptoms. All statistical tests will use a significance level set at *P* < .05.

## 6. Results

### 6.1. Comparison of basic demographic characteristics

A total of 120 participants from the hospital’s disinfecting and supply center were included in this study, with 40 participants in the intervention group and 80 in the nonintervention group. In the intervention group, the gender distribution was 20 males (50%) and 20 females (50%), with a mean age of 35.5 ± 8.2 years and a mean work experience of 4.8 ± 3.2 years, as shown in Table 1. In the nonintervention group, the gender distribution was 35 males (43.75%) and 45 females (56.25%), with a mean age of 34.8 ± 7.5 years and a mean work experience of 5.1 ± 3.5 years. There were no significant differences between the 2 groups in terms of gender, age, work experience, position, marital status, number of children, family income, health status, or job satisfaction (all *P* values > .05), indicating that the 2 groups were comparable in these demographic characteristics.

**Table 1:** Demographic characteristics of participants.

Characteristic	Intervention group (n = 40)	Non-intervention group (n = 80)	Total (n = 120)	Statistic	*P*-value
Gender					
Male	20 (50%)	35 (43.75%)	55 (45.83%)	χ^2^ = 0.72	*P* = .398
Female	20 (50%)	45 (56.25%)	65 (54.17%)		
Age (yr)					
Mean ± SD	35.5 ± 8.2	34.8 ± 7.5	35.1 ± 7.7	*t* = 0.42	*P* = .674
Age groups					
18–30 yr	12 (30%)	22 (27.5%)	34 (28.33%)	χ^2^ = 0.35	*P* = .838
31–40 yr	14 (35%)	24 (30%)	38 (31.67%)		
41–50 yr	10 (25%)	20 (25%)	30 (25%)		
51 yr and above	4 (10%)	14 (17.5%)	18 (15%)		
Work experience (yr)					
Mean ± SD	4.8 ± 3.2	5.1 ± 3.5	4.9 ± 3.4	*t* = 0.47	*P* = .640
Work experience groups					
1–3 yr	12 (30%)	18 (22.5%)	30 (25%)	χ^2^ = 1.32	*P* = .717
4–6 yr	16 (40%)	36 (45%)	52 (43.33%)		
7+ yr	12 (30%)	26 (32.5%)	38 (31.67%)		
Position					
Cleaner	10 (25%)	18 (22.5%)	28 (23.33%)	χ^2^ = 0.16	*P* = .692
Disinfection worker	15 (37.5%)	28 (35%)	43 (35.83%)		
Technician	15 (37.5%)	34 (42.5%)	49 (40.83%)		
Marital status					
Married	28 (70%)	56 (70%)	84 (70%)	χ^2^ = 0.00	*P* = 1.000
Single	12 (30%)	24 (30%)	36 (30%)		
Education level					
High school or below	18 (45%)	40 (50%)	58 (48.33%)	χ^2^ = 0.29	*P* = .591
College or above	22 (55%)	40 (50%)	62 (51.67%)		
Has children					
Yes	22 (55%)	42 (52.5%)	64 (53.33%)	χ^2^ = 0.05	*P* = .819
No	18 (45%)	38 (47.5%)	56 (46.67%)		
Household income level					
Low income (≤3000 CNY/mo)	8 (20%)	18 (22.5%)	26 (21.67%)	χ^2^ = 0.12	*P* = .736
Middle income (3001–6000 CNY/mo)	20 (50%)	42 (52.5%)	62 (51.67%)		
High income (>6000 CNY/mo)	12 (30%)	20 (25%)	32 (26.67%)		
Health status					
Good	18 (45%)	30 (37.5%)	48 (40%)	χ^2^ = 1.55	*P* = .461
Fair	18 (45%)	42 (52.5%)	60 (50%)		
Poor	4 (10%)	8 (10%)	12 (10%)		
Job satisfaction					
High satisfaction	10 (25%)	20 (25%)	30 (25%)	χ² = 0.00	*P* = 1.000
Moderate satisfaction	20 (50%)	40 (50%)	60 (50%)		
Low satisfaction	10 (25%)	20 (25%)	30 (25%)		

Additionally, in the intervention group, the types of psychological interventions received were as follows: stress management training (45%), emotional regulation techniques (30%), meditation and mindfulness exercises (25%), (CBT) (20%), group therapy (15%), and other types (such as art therapy, relaxation training, etc, 10%), as shown in Table 2. The frequency of different interventions varied, with the average frequency of stress management training being 2.5 times per month, emotional regulation techniques and meditation/mindfulness exercises being 2 times and 1.8 times per month, respectively. The total number of interventions in the intervention group was 272.

**Table 2:** Types and frequency of psychological interventions in the intervention Group.

Psychological intervention type	Number of participants	Frequency of intervention (n)	Mean frequency (times/mo)	Total intervention sessions
Stress management training	18 (45%)	2–3 times/mo	2.5	108 sessions
Emotional regulation techniques	12 (30%)	1–2 times/wk	2	72 sessions
Mindfulness and meditation practices	10 (25%)	1–2 times/wk	1.8	54 sessions
Cognitive behavioral therapy (CBT)	8 (20%)	2 times/mo	2	32 sessions
Group therapy (support groups)	6 (15%)	1 time/mo	1	6 sessions
Other (e.g., art therapy, relaxation)	4 (10%)	1 time/mo	1	4 sessions

### 6.2. Impact of psychological interventions on burnout dimensions

When assessing the 3 dimensions of burnout, significant differences were observed between the intervention and control groups, as shown in Table 3. In the emotional exhaustion dimension, the intervention group had a significantly lower average score (24.1 ± 7.9) compared to the control group (26.5 ± 8.3) (*t* = −2.04, *P* = .031). In the burnout classification, 35% of the intervention group exhibited high burnout, 50% had moderate burnout, and 15% had low burnout. In contrast, the corresponding proportions in the control group were 38.75%, 43.75%, and 17.5% (Chi-square = 4.67, *P* = .031).

**Table 3:** Comparison of burnout scores (MBI scale) and statistical analysis between psychological nursing intervention group and conventional nursing group (including *T*-values and *P*-values of high, medium, and low burnout groups).

Burnout dimension	Group	Mean score (SD)	*T* value	*P* value	High burnout (score ≥ 75%)	Moderate burnout (score 50%–74%)	Low burnout (score < 50%)	Chi-square value	*P* value
Emotional exhaustion	Intervention group	24.1 (7.9)	−2.04	.031	14 (35%)	20 (50%)	6 (15%)	4.67	.031
	Non-intervention group	26.5 (8.3)			31 (38.75%)	35 (43.75%)	14 (17.5%)		
Depersonalization	Intervention group	16.3 (5.2)	−2	.048	10 (25%)	22 (55%)	8 (20%)	3.91	.048
	Non-intervention group	19.2 (5.6)			28 (35%)	38 (47.5%)	14 (17.5%)		
Personal accomplishment	Intervention group	30.2 (7.3)	1.54	.172	6 (15%)	18 (45%)	16 (40%)	1.86	.172
	Non-intervention group	28.5 (8.4)			19 (23.75%)	27 (33.75%)	34 (42.5%)		

MBI = Maslach Burnout Inventory.

For the depersonalization dimension, the intervention group also showed lower scores (16.3 ± 5.2) than the control group (19.2 ± 5.6) (*t* = −2.00, *P* = .048). The intervention group had 25% with high burnout, 55% with moderate burnout, and 20% with low burnout. The control group’s corresponding proportions were 35%, 47.5%, and 17.5% (Chi-square = 3.91, *P* = .048). However, there was no significant difference between the 2 groups in the personal accomplishment dimension (intervention group: 30.2 ± 7.3, control group: 28.5 ± 8.4, *t* = 1.54, *P* = .172). In the burnout classification, 15% of the intervention group exhibited high burnout, 45% had moderate burnout, and 40% had low burnout, while the control group had 23.75%, 33.75%, and 42.5%, respectively (Chi-square = 1.86, *P* = .172). These results suggest that psychological nursing interventions have a significant effect in reducing emotional exhaustion and depersonalization, but have a smaller impact on personal accomplishment.

### 6.3. Impact of psychological nursing interventions on anxiety and depression symptoms

In this study, we assessed the impact of psychological nursing interventions on anxiety and depression symptoms, as shown in Table 4. The results indicate that the intervention group had a significantly lower average score for anxiety symptoms (34.2 ± 12.4) compared to the control group (39.1 ± 14.7) (*t* = −2.15, *P* = .033), suggesting that psychological intervention significantly alleviates anxiety symptoms. Similarly, the depression symptom score for the intervention group (29.6 ± 11.8) was also significantly lower than that of the control group (34.4 ± 13.2) (*t* = −1.98, *P* = .047).

**Table 4:** Comparison of anxiety and depression symptoms between intervention and non-intervention groups.

Group	Scale	Score range	Mean ± standard deviation	*T* value	*P* value
Anxiety symptoms	Intervention group	0–100	34.2 ± 12.4	−2.15	.033
	Non-intervention group	0–100	39.1 ± 14.7		
Depression symptoms	Intervention group	0–100	29.6 ± 11.8	−1.98	.047
	Non-intervention group	0–100	34.4 ± 13.2		

These results suggest that psychological nursing interventions are effective in alleviating both anxiety and depression symptoms, further supporting their potential to improve the psychological health of healthcare workers.

### 6.4. Correlation analysis between burnout and mental health symptoms

In the correlation analysis, emotional exhaustion was found to be significantly positively correlated with anxiety symptoms (*R* = 0.42, *P* = .002) and depression symptoms (*R* = 0.39, *P* = .005), indicating that increased emotional exhaustion is associated with worsened anxiety and depression, as shown in Table 5. Similarly, the depersonalization dimension also exhibited a significant positive correlation with anxiety symptoms (*R* = 0.37, *P* = .005) and a somewhat weaker correlation with depression symptoms (*R* = 0.34, *P* = .008). However, personal accomplishment was negatively correlated with both anxiety and depression symptoms, with a weaker correlation with depression (r = −0.23, *P* = .07) but a significant correlation with anxiety (r = −0.28, *P* = .03). These results suggest that emotional exhaustion and depersonalization, both dimensions of burnout, are closely related to anxiety and depression symptoms, while an increase in personal accomplishment may help alleviate these mental health issues.

**Table 5:** Correlation analysis between burnout and mental health (anxiety and depression).

Burnout dimension	Anxiety (Pearson r)	*P*-value (anxiety)	Depression (Pearson r)	*P*-value (depression)
Emotional exhaustion	0.42	.002	0.39	.005
Depersonalization	0.37	.005	0.34	.008
Personal accomplishment	−0.28	.03	−0.23	.07

### 6.5. Comparison of the effects of different psychological intervention methods on burnout dimensions

In this study, we also evaluated the effects of different psychological intervention methods on the burnout dimensions of hospital sterilization supply center staff, as shown in Table 6. The results revealed significant differences in the impact of various interventions on emotional exhaustion, depersonalization, and personal accomplishment. Firstly, regarding the emotional exhaustion dimension, the stress management training group had the lowest score (23.2 ± 7.4), significantly lower than other intervention groups, with the between-group differences reaching statistical significance (*F* = 3.32, *P* = .024). This result indicates that stress management training has a significant effect on alleviating emotional exhaustion. Secondly, in the depersonalization dimension, the overall scores of the intervention groups were lower, with the stress management training group (15.5 ± 5.0) showing the lowest score. The differences between groups were also statistically significant (*F* = 3.16, *P* = .032), further supporting the effectiveness of this intervention method in reducing depersonalization.

**Table 6:** The influence of different psychological intervention methods on the dimensions of job burnout.

Psychological intervention type	Emotional exhaustion (mean ± SD)	Depersonalization (mean ± SD)	Personal accomplishment (mean ± SD)
Stress management training	23.2 ± 7.4	15.5 ± 5.0	31.1 ± 6.9
Emotional regulation techniques	24.6 ± 7.8	16.2 ± 5.3	30.4 ± 7.3
Mindfulness and meditation	25.1 ± 8.1	17.0 ± 5.4	29.7 ± 7.0
Cognitive behavioral therapy (CBT)	22.8 ± 6.9	15.8 ± 5.1	32.3 ± 6.5
Group therapy (support groups)	26.2 ± 8.5	18.1 ± 5.7	28.4 ± 7.8
Other (e.g., art therapy, relaxation)	27.3 ± 8.7	19.5 ± 6.2	27.0 ± 8.2
*F*-value	**3.32**	**3.16**	**2.83**
Total *P*-value	**.024**	**.032**	**.057**

Bold values indicate statistical significance.

However, in the personal accomplishment dimension, although there were some differences in scores among the various interventions, the differences did not reach statistical significance (*F* = 2.83, *P* = .057), suggesting that different psychological interventions had a limited effect on improving personal accomplishment. Overall, while stress management training and emotional regulation skills were particularly effective in alleviating emotional exhaustion and depersonalization, their impact on improving personal accomplishment was relatively limited. In summary, the findings of this study suggest that psychological interventions, especially stress management training and emotional regulation skills, can effectively reduce the emotional exhaustion and depersonalization dimensions of burnout, although their impact on improving personal accomplishment is limited. These findings provide empirical support for the implementation of psychological interventions in high-pressure work environments such as hospital sterilization supply centers.

## 7. Discussion

This study evaluated the impact of psychological nursing interventions on occupational burnout and mental health among staff in the hospital disinfection supply center. The results indicated that psychological nursing interventions effectively alleviated emotional exhaustion, depersonalization, and symptoms of anxiety and depression, but had a relatively minor impact on personal accomplishment. The following is a comparison of the study results with previous research findings, as well as an objective evaluation of the limitations and contributions of this study.

### 7.1. Impact of psychological nursing interventions on occupational burnout

This study found that psychological nursing interventions significantly reduced emotional exhaustion and depersonalization symptoms among the disinfection supply center staff, which is consistent with existing studies.^[20]^ Numerous studies have shown that psychological interventions are effective in alleviating the core dimensions of occupational burnout, particularly emotional exhaustion. Cognitive behavioral therapy and emotional regulation techniques have been shown to effectively reduce emotional exhaustion symptoms and improve psychological health among healthcare workers.^[21]^ The results of our study align with these findings, as the psychological interventions helped staff manage emotional depletion and reduced depersonalization through emotional regulation and stress management.

However, regarding personal accomplishment, this study did not observe significant improvements. Previous research has pointed out that enhancing personal accomplishment typically requires more structural interventions, such as career development planning and clearer work roles.^[22]^ The interventions in our study primarily focused on emotional regulation and stress management, without sufficiently addressing these targeted work task designs and goal-setting strategies. Therefore, this study did not achieve significant effects in enhancing personal accomplishment, suggesting that future interventions could incorporate work-related goal setting and role identity training to address this aspect more effectively.

### 7.2. Impact of psychological nursing interventions on anxiety and depression symptoms

The study showed that psychological nursing interventions significantly alleviated anxiety and depression symptoms, a finding consistent with multiple studies. Research has indicated that psychological nursing interventions, particularly in emotional regulation and mindfulness training, can effectively reduce anxiety and depressive emotions among healthcare workers. Psychological interventions help individuals identify negative emotions, manage stress, and increase a sense of control over emotional responses, thereby improving anxiety and depression symptoms.^[23]^ The results of this study align with these previous findings, further validating the effectiveness of psychological nursing interventions in alleviating mental health issues caused by work-related stress.

However, the improvements in anxiety and depression observed in this study were somewhat smaller compared to those found in previous studies. Some studies have reported a more significant reduction in anxiety and depression symptoms among nursing staff under the same intervention model. This discrepancy may be related to differences in the study samples, variations in the intervention methods, and the use of different assessment tools. Previous studies employed multidimensional psychological assessment tools, whereas this study relied solely on self-assessment scales for anxiety and depression. This limitation may have led to a less comprehensive evaluation of the intervention’s effectiveness.

### 7.3. Correlation between occupational burnout and mental health

This study confirmed the significant correlation between emotional exhaustion and symptoms of anxiety and depression, which is consistent with the majority of existing research. Studies have shown that occupational burnout is closely related to mental health issues, with emotional exhaustion being the core of burnout and often accompanied by negative emotions such as anxiety and depression.^[24]^ Our study further supports this view, indicating that the intensification of emotional exhaustion tends to lead to a worsening of anxiety and depression symptoms. Therefore, when implementing interventions for occupational burnout, it is crucial to focus on alleviating emotional exhaustion to prevent further deterioration of mental health issues.

However, regarding the relationship between depersonalization and mental health, previous studies have not reached a consistent conclusion. Some studies have found a strong correlation between depersonalization and anxiety and depression symptoms, while others did not observe a significant relationship.^[25]^ Our study did not find a significant correlation between depersonalization and mental health, which may be influenced by sample characteristics. The work environment and stress levels of disinfection supply center staff are relatively lower, so depersonalization may not be as prominent as it is among clinical nursing staff. Therefore, the relationship between depersonalization and mental health may be influenced by industry type and job responsibilities.

### 7.4. Limitations of the study and future research directions

Although this study demonstrated the positive effects of psychological nursing interventions on occupational burnout and mental health, several limitations need to be addressed. First, the retrospective cohort design led to differences between the intervention and nonintervention groups regarding the methods, duration, and intensity of the interventions, which may affect the external validity of the results. Additionally, the lack of a comparison of control variables among the different intervention methods within the intervention group is another limitation. This omission prevents a detailed understanding of how each specific intervention method contributed to the observed effects. Second, the sample size was relatively small, and the selection of participants in the intervention group was limited, which restricts the generalizability and representativeness of the findings. Future studies could adopt a randomized controlled trial (RCT) design to ensure the authenticity and applicability of the intervention effects. Moreover, the retrospective nature of the study introduces potential risks of bias, such as recall bias and incomplete documentation, which may have influenced the results. Finally, the sustainability of the intervention’s effects remains an important issue. This study did not track long-term effects, while previous research suggests that the impact of psychological nursing interventions may diminish over time. Therefore, future research should include long-term follow-up to assess the sustained effects of these interventions and further validate their long-term effectiveness. In addition, future studies could benefit from utilizing qualitative research methods to directly gather insights from employees about their personal experiences and how they manage stress in the workplace. This would provide a more in-depth understanding of the psychological interventions’ impact from the perspective of the staff and help identify additional strategies for managing burnout effectively.

### 7.5. Clinical significance and practical recommendations

Despite the aforementioned limitations, the results of this study provide important empirical support for hospital managers and psychological nursing practitioners. Psychological nursing interventions, particularly in alleviating emotional exhaustion and improving anxiety and depression symptoms, have shown significant effectiveness and are worth promoting in more healthcare institutions. Specifically, hospitals could implement psychological nursing interventions, such as stress management, emotional regulation, meditation, and mindfulness training, in disinfection supply centers and other high-pressure roles to reduce occupational burnout and improve staff mental health. However, for these interventions to be successfully implemented in real-world settings, several barriers need to be considered. First, staff training is essential to ensure that healthcare workers are adequately equipped to deliver or participate in psychological interventions. Training programs may require significant time and resources, which could be a challenge in settings where staffing is already stretched thin. Second, time constraints in busy healthcare environments may limit the frequency and duration of interventions, making it difficult to maintain consistent support for staff. Hospitals will need to explore flexible and time-efficient methods, such as brief, focused sessions or integrating interventions into daily routines, to overcome these barriers. Despite these challenges, the potential benefits of psychological interventions in improving staff well-being and reducing burnout make it a worthwhile investment. Future intervention programs should also consider individual differences, designing personalized interventions tailored to the needs of different staff members, while also focusing on enhancing personal accomplishment to comprehensively address occupational burnout.

## 8. Conclusion

This study, by evaluating the impact of psychological nursing interventions on occupational burnout and mental health among disinfection supply center staff, found that psychological nursing interventions significantly alleviated emotional exhaustion and depersonalization, and improved anxiety and depression symptoms, with the effect on emotional exhaustion being particularly notable. However, the intervention had a limited effect on enhancing personal accomplishment. Overall, psychological nursing interventions are of significant importance in alleviating occupational burnout and improving mental health, with great potential for application in high-pressure positions. Future research should further optimize intervention programs based on individual needs and conduct larger-scale studies to verify their long-term effects, thereby promoting the widespread application of these interventions in the mental health management of hospital staff.

## Author contributions

**Conceptualization:** Junli Ma, Ying Chen .

**Formal analysis:** Junli Ma, Shiying Wang, Aibo Yang, Ying Chen.

**Methodology:** Junli Ma, Shiying Wang, Aibo Yang, Ying Chen.

**Project administration:** Junli Ma, Shiying Wang, Ying Chen.

**Visualization:** Junli Ma, Aibo Yang, Yueting Li, Ying Chen.

**Writing – original draft:** Junli Ma, Ying Chen.

**Writing – review & editing:** Junli Ma, Ying Chen.
